# Efficient subtraction of insect rRNA prior to transcriptome analysis of *Wolbachia-Drosophila* lateral gene transfer

**DOI:** 10.1186/1756-0500-5-230

**Published:** 2012-05-14

**Authors:** Nikhil Kumar, Todd Creasy, Yezhou Sun, Melissa Flowers, Luke J Tallon, Julie C Dunning Hotopp

**Affiliations:** 1 Institute for Genome Sciences, University of Maryland School of Medicine, Baltimore, MD 21201, USA; 2 Department of Microbiology and Immunology, University of Maryland School of Medicine, Baltimore, MD 21201, USA

**Keywords:** *Wolbachia*, *Drosophila ananassae*, RNASeq, Transcriptomics, Lateral gene transfer, Horizontal gene transfer, Endosymbiont, Insect vector

## Abstract

**Background:**

Numerous methods exist for enriching bacterial or mammalian mRNA prior to transcriptome experiments. Yet there persists a need for methods to enrich for mRNA in non-mammalian animal systems. For example, insects contain many important and interesting obligate intracellular bacteria, including endosymbionts and vector-borne pathogens. Such obligate intracellular bacteria are difficult to study by traditional methods. Therefore, genomics has greatly increased our understanding of these bacteria. Efficient subtraction methods are needed for removing both bacteria and insect rRNA in these systems to enable transcriptome-based studies.

**Findings:**

A method is described that efficiently removes >95% of insect rRNA from total RNA samples, as determined by microfluidics and transcriptome sequencing. This subtraction yielded a 6.2-fold increase in mRNA abundance. Such a host rRNA-depletion strategy, in combination with bacterial rRNA depletion, is necessary to analyze transcription of obligate intracellular bacteria. Here, transcripts were identified that arise from a lateral gene transfer of an entire *Wolbachia* bacterial genome into a *Drosophila ananassae* chromosome. In this case, an rRNA depletion strategy is preferred over polyA-based enrichment since transcripts arising from bacteria-to-animal lateral gene transfer may not be poly-adenylated.

**Conclusions:**

This enrichment method yields a significant increase in mRNA abundance when poly-A selection is not suitable. It can be used in combination with bacterial rRNA subtraction to enable experiments to simultaneously measure bacteria and insect mRNA in vector and endosymbiont biology experiments.

## Findings

### Background

Many interesting bacteria form intimate, obligate relationships with eukaryotes. These bacteria include endosymbionts and obligate intracellular pathogens. These microbes can be difficult to research as they cannot be cultured, easily manipulated, or genetically transformed. Therefore, genomics techniques have significantly advanced the study of these organisms. Because of the obvious potential health impacts, many techniques and tools have been developed to research such bacteria that interact with humans. For example, the Ribo-Zero rRNA removal kit for human/mouse/rat (Epicentre, Madison, WI, USA) can facilitate transcriptome analysis of intracellular pathogens of humans. However, for non-mammalian systems including bacteria/vector systems, a void still exists. Previously, the MICROBEnrich insect/*C. elegans* module (Ambion, Austin, TX, USA) was used for this purpose, but it is no longer available. Therefore, we sought to investigate if Epicentre’s Ribo-Zero rRNA removal kit designed for humans and rodents would efficiently remove rRNA from insect samples. *Drosophila ananassae* is a fruit fly that can be naturally infected with a *Wolbachia* endosymbiont [1,2]. In addition, some *Wolbachia*-colonized lines have an entire *Wolbachia* genome transferred to a fly nuclear chromosome [3]. Transcripts from nuclear *Wolbachia* transfers (nuwts) are of particular interest, as they can be used to elucidate functions for the nuwts. Previously, we identified 28 nuwt transcripts (~2% of the *Wolbachia* genome) in adult flies, albeit at low levels [3]. Since these transcripts might not be polyadenylated, poly-A enrichment of total RNA is not a suitable technique for obtaining mRNA prior to RNASeq. Therefore, Ribo-Zero was tested as a suitable alternative.

### RNA isolation, mRNA enrichment, and transcriptome sequencing

Total RNA was isolated with TRIzol, as previously described [3], from 50–85 freshly laid eggs of wild-type *D. ananassae* In(3R)A (Stock No. 14024–0371.13) that is infected with the *Wolbachia* endosymbiont *w*Ana [2] and from a tetracycline-treated (*Wolbachia*-cured) line of this fly. Both lines are tested regularly by fluorescence *in situ* hybridization to confirm the presence or absence of a *Wolbachia* infection, respectively. The samples were enriched for mRNA using the Ribo-Zero rRNA removal kit for human/mouse/rat (Epicentre, Madison, WI, USA), following the manufacturer’s protocol.

### Assessment of rRNA removal by microfluidics

**Figure 1:**
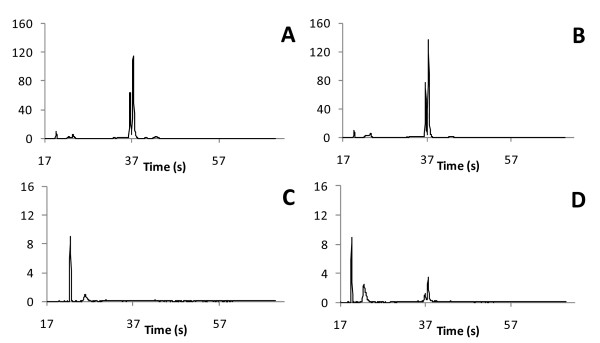
**Bioanalyzer analysis of Ribo-Zero-subtracted RNA.** The subtraction of rRNA using the Ribo-Zero human/mouse/rat reagents was tested on total RNA from *Drosophila ananassae* with (panels **A** and **C**) and without (panels **B** and **D**) the presence of its *Wolbachia* endosymbiont. The total RNA prior to Ribo-Zero subtraction (panels **A** and **B**) is compared to RNA after Ribo-Zero subtraction (panels **C** and **D**). The starting amount of RNA prior to subtraction for panels **C** and **D** was equivalent to the amount shown in panels **A** and **B**.

Comparison of the original and Ribo-Zero-subtracted samples on a Bioanalyzer (Agilent, Santa Clara, CA, USA) illustrates the efficient removal of the majority of the rRNA (Figure 1) and the loss of >97% of the RNA, as assessed by integrating the area under the rRNA peaks. In *D. ananassae*, as well as in many insects, the 28S rRNA is naturally cleaved resulting in two peaks that are unusually close together on the Bioanalyzer when compared to other eukaryotic or bacterial rRNA samples. Importantly, the Ribo-Zero subtraction efficiently removed both halves of the cleaved rRNA.

### Assessment of rRNA removal by transcriptome sequencing

**Figure 2:**
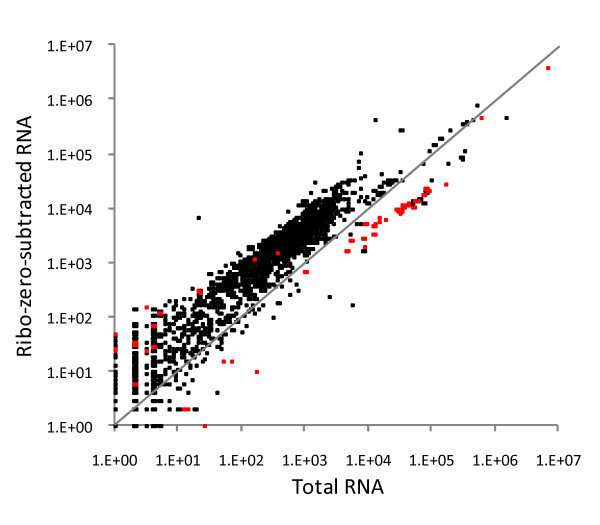
**Comparison of total RNA and Ribo-Zero-subtracted RNA from *Drosophila ananassae.*** The reads from both samples were mapped with BWA against the *D. ananassae* scaffolds. Each scaffold was then computationally divided into 100 kbp fragments and the number of reads mapping to the fragment were counted. When scaffolds were <100 kbp, the entire scaffold was counted. The last fragment of each scaffold was always <100 kbp. Fragments containing annotated rRNA are shifted right of the diagonal (gray line) due to decreased representation in the Ribo-Zero-treated RNA. Meanwhile, fragments without rRNA are shifted leftward of the diagonal (gray line) because of their increased abundance relative to the entire sequenced population in the subtracted samples.

While the Bioanalyzer is highly sensitive at detecting intact rRNA, degraded rRNA can go undetected. Therefore, we also sought to examine RNASeq results to ensure even subtraction across the rRNA. Paired-end libraries (Illumina, San Diego, CA, USA) were constructed using the two pools of RNA for the cured line (Figure 1B and 1D) using the standard protocol starting immediately after the poly-A selection step. Half of a channel of 72-bp reads was obtained on a GAIIx (Illumina, San Diego, CA, USA) for each library. The sequencing reads were mapped against the reference *D. ananassae* assembly, using the default parameters for BWA [4], yielding 15.8 million and 15.0 million mapped reads from total RNA and the Ribo-Zero-subtracted sample, respectively. The reads for each 100 kbp window across the genome are plotted, comparing the subtracted sample from the total RNA (Figure 2). In this subtraction, and unlike the subtraction with the RNA from uncured specimens, the Bioanalyzer revealed that only 98% of rRNA was removed. Therefore, and as expected, a significant amount of rRNA is still present, as illustrated by the red dots representing fragments with at least a portion of an rRNA. However, the results are consistent with removal of >90% of the rRNA with a shift leftward of the majority of 100 kbp fragments due to enrichment for mRNA and separation of the rRNA from the mRNA by approximately one order of magnitude.

**Figure 3:**
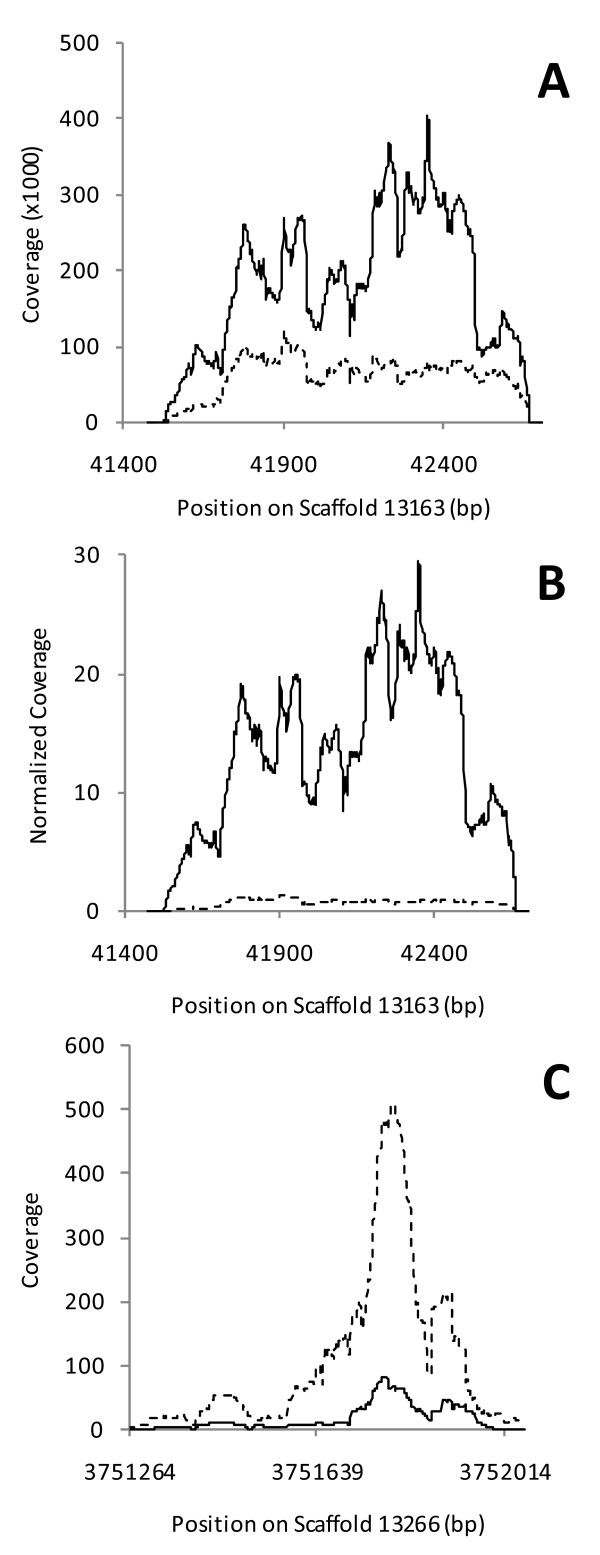
**Coverage of the 18S rRNA and actin genes.** The coverage of reads across the 18S rRNA (Panel A) and actin (Panel C) genes was determined by mpileup in samtools and compared between total RNA (solid line) and Ribo-Zero-subtracted RNA (dashed line). For the 18S rRNA, the Ribo-Zero-subtracted sample contained 66% fewer rRNA reads when compared to the total RNA sample. When the 18S rRNA results are normalized relative to actin, a 95% reduction in the rRNA is seen (Panel B). The 18S rRNA is only partially sequenced in the reference genome with a gap in the scaffold to the immediate right of this region.

While the overall trend is clear, specific points may not reflect this trend. For example, the 100 kbp region containing the 18S rRNA is the red point in the upper right corner that appears unshifted from the diagonal. In the total RNA, 2,936,794 reads mapped to the 18S rRNA fragment (41,477-43,516 bp on scaffold 13163; GenBank CH902719.1); in the subtracted sample 980,094 reads (66%) were sequenced (Figure 3A). This would be sufficient to offset the point from diagonal except that an additional ~3 million reads map in this 100 kbp fragment and outside the 18S rRNA. These reads prevent the full shift of this point relative to the diagonal that would be realized should only the 18S rRNA be present in this fragment.

While there is a 66% difference in the raw number of 18S rRNA reads between the two samples (Figure 3A), this does not fully capture the Ribo-Zero subtraction. Subtraction of rRNA means that significantly more reads are obtained from the remaining RNA species in the subtracted sample, as seen in the 6.2-fold increase in signal for actin in the subtracted sample (Figure 3C). When the rRNA results for each sample are normalized to the number of reads in actin, a 95% reduction of rRNA is observed (Figure 3B). This is consistent with the 98% reduction determined by integrating the area under the peak on the Bioanalyzer.

Not only does the subtraction increase the signal for genes as shown above with actin, it also increases the number of genes that can be analyzed. While 8,888 transcripts had at least a single read mapping in the subtracted sample, only 7,629 transcripts had a single read mapping in the unsubtracted sample. Yet, a single read is not very informative when examining differential expression, and instead, a minimum number of reads/transcript may be required for a differential expression analysis. Standards for this minimum have not been established to our knowledge. But if one required 100 reads/transcript, the subtracted sample would have 3,677 transcripts that could be analyzed while the unsubtracted sample would only have 1,047 transcripts.

### Detection of transcripts arising from bacteria-to-animal lateral gene transfer

While the rRNA was sufficiently removed from the studied samples, only a few reads (19 and 20 reads from the total RNA and the Ribo-Zero-subtracted RNA, respectively) arose from transcripts of nuwts as identified by mapping with BWA [4] against the wRi reference genome [5] (Table 1). No transcripts were identified with more than one mapped read after duplicate removal. This low abundance of reads is not sufficient for analysis of the nuwt transcriptome. There was no overlap in the reads found between the two samples with the exception of reads that likely arose from non-*Wolbachia* bacterial contaminants on the surface of the eggs, further suggesting the stochastic detection of these low abundance transcripts.

**Table 1:** ***Wolbachia*genes represented in reads and arising from nuwts**

				**Pairs of Reads**		**Unpaired Reads**	
**5′-end**	**3′-end**	**Locus**	**Name**	**Total RNA**	**Ribo-Zero-subtracted**	**Total RNA**	**Ribo-Zero-subtracted**
8305	10323	WRi_000090	type IV secretion system protein VirD4	0	2*	0	0
20819	29332	WRi_000230	DNA-directed RNA polymerase, beta subunit	0	1	0	0
37758	35593	WRi_000280	ankyrin repeat domain protein	0	1	0	0
71725	72540	WRi_000680	hypothetical protein	1	0	0	0
188532	191277	WRi_r01850	23S ribosomal RNA	0	0	1‡	0
458087	457221	WRi_004260	4-diphosphocytidyl-2 C-methyl-D-erythritol kinase	0	1	0	0
663596	664402	WRi_006160	hypothetical protein	1	0	0	0
687296	685893	WRi_006360	transcription termination factor Rho	0	1	0	0
804498	806600	WRi_007420	hypothetical protein	3*	0	0	0
832611	833609	WRi_007700	tryptophanyl-tRNA synthetase	0	1	0	0
938372	936990	WRi_008690	transposase	2*^,†^	0		
1044899	1045516	WRi_009720	lipoyltransferase	0	0	1	0
1139870	1138959	WRi_010540	transcriptional regulator, putative	0	1†	0	0
1289969	1291473	WRi_r11990	16S ribosomal RNA	0	0	1‡	2‡
1334777	1336324	WRi_012430	penicillin-binding protein	1	0	0	0
1336345	1337274	WRi_012440	4-hydroxy-3-methylbut-2-enyl diphosphate reductase	1	0	0	0
1436571	1435714	WRi_013460	4-hydroxybenzoate octaprenyltransferase	0	1	0	0

^*^These are duplicate paired end reads; upon manually removing duplicates this reduces to one. However, these reads were not removed when analyzed with the duplicate analysis tool Picard.

^†^These reads are not unique with respect to the reference genome.

^‡^These reads likely arise from non-*Wolbachia* bacterial contaminants on the eggs.

This low abundance of transcription mirrors previous findings that transcription of nuwts is low [3]. In previous work, nuwts were found to be 10^4^-10^7^ times less abundant than actin in adult flies [3], which is consistent with the RNASeq results presented here for eggs. This level of transcription may or may not be biologically relevant. Important tissue-, condition-, and/or stage-specific transcription cannot be ruled out. However the tissue, condition, and/or stage that should be examined are not immediately obvious in the absence of a phenotype.

## Conclusions

The Ribo-Zero rRNA removal kit for human/mouse/rat efficiently removes >98% of insect rRNA from total RNA samples, yielding a 6.2-fold increase in detection of mRNA transcripts. In this data, 3× as many transcripts can be evaluated in a differential gene expression analysis requiring at least 100 reads/transcript. Coupling this Ribo-Zero kit with ones designed for removal of bacterial rRNA would also easily reduce endosymbiont rRNA and allow the concomitant sequencing of host and bacterial mRNA from endosymbiont containing samples.

## Availability of supporting data

The sequencing reads supporting the results of this article are available in the sequence read archive, SRA045520 at http://www.ncbi.nlm.nih.gov/sra?term=SRA045520.

## Abbreviations

nuwt: Nuclear Wolbachia transfer.

## Competing interests

The authors declare that they have no competing interests.

## Authors’ contributions

NK reared the flies including curing with tetracycline, isolated RNA for the experiments, performed the RNA subtractions, and drafted portions of the manuscript. TC, YS, and LJT analyzed the data and generated figures. MF and LJT were responsible for sequencing the RNA. JCDH conceived of the study, participated in its design and coordination, and drafted the manuscript. All authors read and approved the final manuscript.
